# Differential Excretory/Secretory Proteome of the Adult Female and Male Stages of the Human Blood Fluke, *Schistosoma mansoni*

**DOI:** 10.3389/fpara.2022.950744

**Published:** 2022-07-18

**Authors:** Eric T. Kenney, Victoria H. Mann, Wannaporn Ittiprasert, Bruce A. Rosa, Makedonka Mitreva, Bethany K. Bracken, Alex Loukas, Paul J. Brindley, Javier Sotillo

**Affiliations:** ^1^Department of Microbiology, Immunology & Tropical Medicine, and Research Center for Neglected Diseases of Poverty, School of Medicine and Health Sciences, George Washington University, Washington, DC, United States; ^2^Department of Internal Medicine, Washington University of St. Louis School of Medicine, St. Louis, MO, United States; ^3^Charles River Analytics, Inc., Cambridge, MA, United States; ^4^Centre for Molecular Therapeutics, Australian Institute of Tropical Health and Medicine, James Cook University, Cairns, QLD, Australia; ^5^Parasitology Reference and Research Laboratory, Centro Nacional de Microbiología, Instituto de Salud Carlos III, Madrid, Spain

**Keywords:** *Schistosoma*, proteomics, secretome, label-free quantification, adults

## Abstract

Intricate molecular communication between schistosome flatworms and their mammalian host, as well as between paired male and female schistosomes has shaped the secreted proteome of these flatworms. Whereas the schistosome egg is responsible for the disease manifestations of chronic schistosomiasis, the long lived, adult female and male stages also release different mediators including glycans, lipids, proteins and small molecules, known as excretory/secretory products (ESPs), that facilitate their survival. Given their importance, deeper analysis focused on analyzing the ESPs from adult schistosomes would likely be informative, beyond current understanding of the complement of ESP proteins. Here, taking advantage of highly accurate and sensitive mass spectrometers, the excretory/secretory proteome from cultured *Schistosoma mansoni* male or female adult worms was identified, quantified, compared and contrasted using a label-free proteomic approach. Approximately 1,000 proteins were identified, from which almost 800 could be quantified. Considering the proteins uniquely identified and proteins with a significantly regulated expression pattern in male or female flukes, a total of 370 and 140 proteins were uniquely or more abundantly secreted by males and females, respectively. Using functional analysis networks showing the gene ontology terms and KEGG pathways with the highest significance, we observed that male schistosomes secrete proteins related to carbohydrate metabolism and cytoskeletal organization more abundantly than females, while female worms secreted more hydrolases and proteins involved in cellular homeostasis than males. This analysis doubles the number of reported excreted/secreted proteins from *S. mansoni*, contributing to deeper understanding of the host-parasite interaction and parasitism. Furthermore, these findings expand potential vaccine and diagnostic candidates for this neglected tropical disease pathogen, and thereby also provide leads for novel intervention to control this disease and its transmission.

## Introduction

Schistosomiasis is a major neglected tropical disease and is considered the most important helminthiasis in terms of morbidity and mortality. More than 200 million people are infected worldwide with 700 million at risk of infection. This remains a major public health problem, particularly in sub-Saharan Africa (Colley et al., 2014; Mcmanus et al., 2018). Human schistosomiasis is caused by six species of blood flukes: *Schistosoma guineensis, S. haematobium, S. intercalatum, S. japonicum, S. mansoni*, and *S. mekongi*. Nowadays, the predominant human species are *S. haematobium* and *S. mansoni*, given the reduction of infection in recent decades caused by *S. japonicum* in the Yangtze River basin provinces of China (Wang et al., 2021). Urogenital schistosomiasis caused by hybrids of *S. haematobium* and *S. bovis* and relatives is spreading in West Africa (Webster et al., 2006; Huyse et al., 2009) and in Corsica (Boissier et al., 2016; Rothe et al., 2021).

Male and female schistosomes dwell in copula within the mesenteric veins (*S. mansoni, S. japonicum*) or the vesical venous plexus (*S*. *haematobium*) of the human, laying hundreds to thousands (depending on the species) of fertilized eggs each day. The eggs traverse the intestinal wall (e.g., *S. mansoni*) or the bladder wall (*S*. *haematobium*) and exit the host to the external environment in feces or urine, respectively. However, many eggs fail to exit the infected person and are retained in host tissues where they induce inflammation, granuloma, and fibrosis (Mcmanus et al., 2018). In the external environment, the eggs hatch when they reach freshwater, each releasing a miracidium, which undergoes cycles of asexual reproduction within the intermediate host (a gastropod snail) shedding thousands of cercariae into the water. After penetrating the skin of the definitive host, the cercaria sheds its tail and the juvenile larva, the schistosomulum, migrates within the circulatory system, reaching the lungs, the liver, and eventually the portal venous system or the venous system that drains the pelvic organs where the fully mature flukes pair and the female produces eggs, completing the developmental cycle.

Whereas the schistosome egg is responsible for the disease manifestations of chronic schistosomiasis, as well as orchestrating the hallmark immunological transition from a Th1 to Th2 response (Pearce and Macdonald, 2002; Schwartz and Fallon, 2018; Acharya et al., 2021), the long lived, adult female and male stages also release mediators that facilitate their intra-vascular existence in a hostile niche where they are bathed in immune cells and effector molecules. These mediators, also known as excretory/secretory products (ESPs), are secreted (or released) from the esophageal gland, the gut epithelium and from the tegument of schistosomes, making it a highly diverse mixture of molecules. The protein composition of the ESPs from several developmental stages and species of schistosomes has been described in depth (Liu et al., 2009; Mathieson and Wilson, 2010; Hall et al., 2011; Dvorak et al., 2016; Sotillo et al., 2016, 2019; Floudas et al., 2017; De Marco Verissimo et al., 2019; Kifle et al., 2020; Neves et al., 2020; Chen et al., 2022), although the diversity, role, and packaging of these secreted and excreted antigens, including as cargo within extracellular vesicles, may not yet be fully characterized or deciphered (Acharya et al., 2021). An early study of adult stage *S. japonicum* ESPs showed the presence of conserved proteins such as metabolic enzymes, heat shock proteins (HSPs), detoxification proteins, and peptidases (Liu et al., 2009). Other studies focusing on the schistosome “vomitus” and other secreted proteins highlighted the presence of different saposins, ferritins and cathepsins, among other molecules (Hall et al., 2011). Furthermore, tetraspanins, annexins, calpain, transporters, and cytoskeletal proteins have been identified on the tegument of *S. mansoni* adult worms (Braschi and Wilson, 2006; Braschi et al., 2006a,b).

Here, the ESPs in cultured supernatants from adult male and adult female *S. mansoni* were isolated and compared using a label-free proteomic approach for the first time, increasing the coverage of the published secretome. About 1,000 proteins were identified, from which ~ 800 could be finally quantified. In sum, this new analysis at least doubles the number of proteins known in these extracts (Hall et al., 2011; Wilson, 2012), substantially expanding the catalog of the protein complement of the ESPs from *S. mansoni*, which provides new insights of the host-parasite interplay. In turn, we augment the number of potential vaccine and diagnostic candidates listed previously for this neglected tropical disease agent.

## Materials and Methods

### Ethics

Mice experimentally infected with *S. mansoni*, obtained from the Schistosomiasis Resource Center (SRC) at the Biomedical Research Institute (BRI), MD were housed at the Animal Research Facility of the George Washington University (GWU), which is accredited by the American Association for Accreditation of Laboratory Animal Care (AAALAC no. 000347) and has an Animal Welfare Assurance on file with the National Institutes of Health, Office of Laboratory Animal Welfare, OLAW assurance number A3205-01. All procedures employed were consistent with the Guide for the Care and Use of Laboratory Animals. The Institutional Animal Care and Use Committee (IACUC) at GWU approved the protocol used for maintenance of mice and recovery of schistosomes.

### Schistosomes

Swiss-Webster albino mice were euthanized seven weeks after infection with *S. mansoni*, livers were removed at necropsy, schistosome eggs recovered from the livers, and adult worms from the portal circulation as described (Dalton et al., 1997).

### Isolation of Adult Excretory/Secretory Products

For the collection of excretory-secretory products (ESPs), adult *S. mansoni* were provided by Schistosomiasis Resource Center (SRC) of the Biomedical Research Institute (BRI), Rockville, MD. The worms were sorted with forceps to separate males (group 1) and females (group 2) or left unpaired (group 3), rinsed briefly in 1 × phosphate buffered saline (PBS) (Corning), each group was then separated into three different batches, and subsequently transferred to tissue culture plates (Sarstedt) and incubated at a density of 13 worms/ml in serum-free Dulbecco's Modification of Eagle's Medium (DMEM) (Corning) supplemented with 2% Antibiotic-Antimycotic (Gibco). Secretion of ESP into the serum-free medium was facilitated by continuous incubation at 37°C, 5% CO_2_ in air (Neves et al., 2020). A total of 20 mL of culture supernatant was removed with minimal disturbance to the schistosomes at 4, 24, 48, 72 h, 5 and 7 days of culture, centrifuged at 400 *g* and 2,000 *g* and stored at −80°C. The drawn medium was retained for storage and was replaced with fresh medium to the culture at each time point. At the conclusion of the collection period, the ESP-containing media were thawed gradually on wet ice, after which ESP was concentrated using Centricon Plus-70 Centrifugal Filter Units (Millipore) featuring a 3 kDa nominal molecular size limit. Concentration by centrifugation on the 3 kDa membrane was undertaken at 3,220 *g* at 4°C using an Eppendorf 5810R centrifuge fitted with an A-4-62 swinging bucket rotor. Concentrated ESP was resuspended and reconcentrated twice using volumes of chilled PBS equivalent to the starting volume of the sample. Protein concentration was ascertained by the Pierce BCA Protein Assay Kit (Thermo Fisher) method, and concentrated ESP was aliquoted and stored at −80°C.

### Mass Spectrometry

For the mass spectrometry analysis, the ESPs from all time points were combined for each sample (male, female or mixed sexes) and analyzed as follows. Three biological replicates of ES from males, females and mixed samples were individually processed as follows. Samples were freeze-dried and dissolved with 22 mL of 50 mM ammonium bicarbonate (ABC). Two (2) mL was used to quantify the protein concentration with Qubit (Invitrogen) reagent according to the manufacturer's instructions. Ten (10) mg of protein was taken and volumes set to 22.5 mL of 50 mM ABC. Reduction and alkylation were performed by incubating samples at 60°C for 20 min with 2 mM dithiothreitol followed by a 30 min incubation at RT in the dark with 5.5 mM 2-iodoacetamide. Thereafter, samples were in-solution digested with 400 ng trypsin overnight at 37°C and acidified with 10% TFA to a final concentration of 1%. Last, digested peptides were concentrated by speed vacuum to 15 μL.

Five (5) μl of peptide mixtures were loaded onto a trap column (3 μm C18-CL, 350 μm ×0.5 mm; Eksigent Technologies, Redwood City, CA) and desalted with 0.1% TFA at 5 μl/min during 5 min. The peptides were then loaded onto an analytical column (3 μm C18-CL 120 Ă, 0.075 ×150 mm; Eksigent) equilibrated in 5% acetonitrile 0.1% FA. Elution was accomplished with a linear gradient of 15–40% B in A for 60 min (A: 0.1% FA; B: ACN, 0.1% FA) at a flow rate of 300 nL/min. Peptides were analyzed in a mass spectrometer nanoESI qQTOF (6,600+ TripleTOF, ABSCIEX). Sample was ionized in a Source Type: Optiflow <1 μL Nano applying 3.0 kV to the spray emitter at 175°C. Analysis was carried out in a data-dependent mode. Survey MS1 scans were acquired from 350–1,400 m/z for 250 ms. The quadrupole resolution was set to “LOW” for MS2 experiments, which were acquired 100–1,500 m/z for 25 ms in “high sensitivity” mode. The following switch criteria were used: charge: 2+ to 4+; minimum intensity; 250 counts per second (cps). Up to 100 ions were selected for fragmentation after each survey scan. Dynamic exclusion was set to 15 s.

### Database Search and Protein Quantification

Database searches were performed using FragPipe (v16.0) with MSFragger (v3.3) (Kong et al., 2017) and Philosopher (v4.0) (Da Veiga Leprevost et al., 2020) against a concatenated target/decoy database consisting of the *S. mansoni* proteome (UP000008854) and common contaminants from Uniprot (downloaded 30 June 2021; 14,615 proteins). For the MSFragger analysis, precursor and fragment mass tolerance were both set to 20 ppm. Mass calibration and parameter optimization were enabled, and isotope error was set to 0/1/2 with two missed trypsin cleavages allowed. The peptide length was set from 7 to 50, and the peptide mass was set to 500–5,000 Da. Carbamidomethylation of C (+57.021464 Da) was set as fixed modification and Oxidation of M (+15.994915 Da) and acetylation of protein N-term (+42.010565 Da) as variable modifications. Philosopher (Da Veiga Leprevost et al., 2020) with PeptideProphet (Keller et al., 2002) and ProteinProphet (Nesvizhskii et al., 2003) was used to estimate the identification FDR. The PSMs were filtered at 1% PSM and 1% protein identification FDR. Quantification and match between runs (MBR) was performed with IonQuant using default values (Yu et al., 2021).

Mass spectrometry data along with the identification results have been deposited in the ProteomeXchange Consortium *via* the PRIDE partner repository (Vizcaino et al., 2013) with the dataset identifier PXD030699.

### Bioinformatic Analysis of Proteomic Sequence Data

Label-free quantitative (LFQ) analysis of identified proteins was performed with the MSstats R package (Choi et al., 2014) using default parameters (equalizeMedians as normalization method; log transformation: 2; Tukey's median polish as the summary method; censored values in intensity column: null and MBimpute: false). Using a power calculation of 0.9 and FDR of 0.05, fold-changes were considered as significant when ≥2.450 and adjusted *p*-value ≤ 0.05. STRINGDB https://string-db.org was used to perform a PPI analysis based on confidence in the interaction (minimum required interaction score ≥0.7) and the network was visualized using Cytoscape. The Cytoscape plugin ClueGO was used to integrate the Kyoto Encyclopedia of Genes and Genomes (KEGG), and Gene Ontology information (including biological processes, immune processes and molecular functions) (Bindea et al., 2009). The enrichment tests for terms and groups were two-sided tests based on the hyper-geometric distribution with a Kappa Score Threshold of 0.4. All GO terms that were significant with *P* <0.05 (after correcting for multiple testing using the Bonferroni step down FDR correction), ranged between 3 and 8 tree intervals and contained a minimum of three genes (representing at least 4% from the total number of associated genes) were selected for further analysis. For each group/cluster, only the node with the smallest adjusted *P*-value was annotated.

The 4,143 *S. mansoni* genes associated with 68 molecularly distinct and annotated clusters identified by single cell RNA-seq profiling (scRNAseq) from an earlier report (Wendt et al., 2020) were retrieved and intersected with the 793 detected ESP proteins. Binomial distribution tests (MS Excel) were used to identify significant enrichment of ESP proteins in each cluster using the total number of ESPs across the clusters as the number of “trials,” the number of ESPs in the specific cluster as the number of “successes” and the proportion of the 4,413 total cluster-enriched genes in the specific cluster as the “expected probability.” FDR correction was applied to the resulting *P*-values for multiple test corrections. A threshold FDR value of 0.01 was considered significant.

## Results

### *S. mansoni* Adult Males Secrete More Proteins in Maintenance Culture Than Adult Female Worms

*S. mansoni* adult worms were obtained from experimentally infected mice and separated into three different groups: males (M), females (F) or left coupled as male and female pairs (MF). The worms were maintained for seven days and the secreted proteins analyzed by LC-MS/MS. A label-free quantitative (LFQ) analysis was performed to identify the proteins with significantly up- or down-regulated abundance in the secretomes of all three sample groups. An input file containing unique and razor peptides for 934 validated proteins was generated by MSFragger (Supplementary Table S1). Data were normalized using medians of summed intensities (Supplementary Figure S1). MSstats was used to estimate the power of the analysis performed. For our analysis, and to have a power calculation = 0.9 and FDR = 0.05, fold-changes were considered as significant when ≥2.450 (Log2 ≥ 1.3) and the adjusted *P*-value ≤ 0.05 (Supplementary Figure S2).

**Figure 1:**
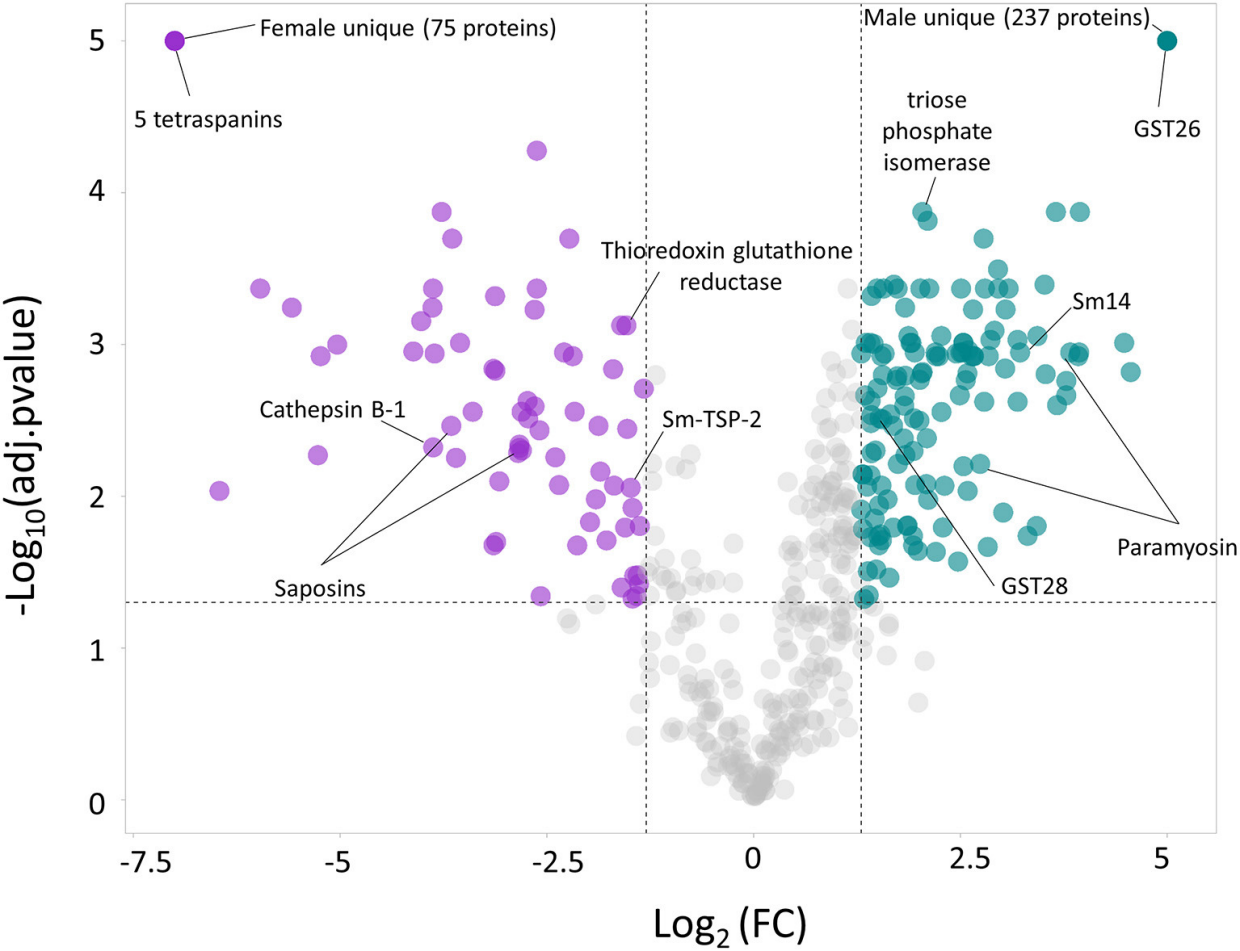
Volcano plot highlighting the proteins with significantly divergent relative protein abundance in the secretome of the male (M) and female (F) *Schistosoma mansoni* flukes. Since uniquely expressed proteins cannot be plotted in a volcano plot, they were assigned arbitrary values of –Log_10_(adj.pvalue) = 5 and Log_2_FC = 5 (males) and Log_2_FC = −7.5 (females).

The quantitative proteomic analysis revealed a clear sex-dependent protein profile. For the M vs. F comparison, a total of 793 proteins were quantified, from which 237 and 75 were uniquely detected in males and females, respectively (Supplementary Table S2). Furthermore, the relative abundance of 133 proteins was significantly higher in the secretome of males (Table 1; Figure 1; Supplementary Table S2), while the abundance level of 65 proteins was significantly higher in the secretome of females (Table 2; Figure 1; Supplementary Table S2).

**Table 1:** The 20 most abundantly secreted proteins by the adult male *Schistosoma mansoni*.

**Protein**	**Annotation**	**Log_**2**_FC**	**Adjusted *P*-value**
TPM1_SCHMA	Tropomyosin-1 (SmTMI)	4.55	0.0015
G4LWI3_SCHMA	Aldehyde dehydrogenase, putative	4.48	0.0009
G4VCN1_SCHMA	Putative fatty acid binding protein	3.94	0.0001
G4VSB7_SCHMA	LIMPETin; Putative four and A half lim domains	3.93	0.0011
G4LX89_SCHMA	Cofilin, actophorin, putative	3.92	0.0011
A0A3Q0KD88_SCHMA	Paramyosin	3.82	0.0011
TPM2_SCHMA	Tropomyosin-2 (SmTMII)	3.78	0.0017
G4M0V2_SCHMA	Transgelin	3.78	0.0021
A0A5K4EZE2_SCHMA	Troponin t, putative	3.66	0.0025
ALF_SCHMA	Fructose-bisphosphate aldolase	3.65	0.0001
G4VJG3_SCHMA	Putative crp1/csrp1/crip1	3.53	0.0015
Q15EU2_SCHMA	Cytochrome c-like protein	3.51	0.0004
A0A3Q0KPD3_SCHMA	Putative EF-hand containing protein	3.43	0.0008
A0A3Q0KF84_SCHMA	Putative myosin light chain 1	3.42	0.0157
SM20_SCHMA	20 kDa calcium-binding protein (Antigen SM20)	3.31	0.0182
FABP_SCHMA	14 kDa fatty acid-binding protein (Sm14)	3.22	0.0011
G4VN13_SCHMA	Uncharacterized protein	3.19	0.0023
A0A3Q0KCV6_SCHMA	Putative troponin I	3.19	0.0009
A0A3Q0KH21_SCHMA	Pyruvate kinase	3.08	0.0004
A0A3Q0KB95_SCHMA	Filamin	3.04	0.0005

**Table 2:** The 20 most abundantly secreted proteins by the adult female *Schistosoma mansoni*.

**Protein**	**Annotation**	**Log_**2**_FC**	**Adjusted *P*-value**
A0A3Q0KC43_SCHMA	Deoxyribonuclease II-related	−6.46	0.0092
A0A3Q0KBJ8_SCHMA	Lysosomal Pro-Xaa carboxypeptidase (S28 family)	−5.97	0.0004
G4VBG5_SCHMA	Ferritin	−5.58	0.0006
A0A5K4FCS2_SCHMA	Cathepsin L	−5.27	0.0054
A0A3Q0KMS2_SCHMA	Uncharacterized protein	−5.24	0.0012
A0A3Q0KF32_SCHMA	Beta-glucosidase	−5.04	0.0010
G4VRT6_SCHMA	Family S28 unassigned peptidase (S28 family)	−4.11	0.0011
G4VRB5_SCHMA	Putative ectonucleotide pyrophosphatase/phosphodiesterase	−4.02	0.0007
G4VBG6_SCHMA	Ferritin	−3.88	0.0006
A0A3Q0KJ08_SCHMA	Putative macroglobulin/complement	−3.88	0.0004
Q8MNY2_SCHMA	Cathepsin B1 isotype 1	−3.87	0.0048
A0A3Q0KN25_SCHMA	Peptidase C1 family	−3.86	0.0011
A0A3Q0KU93_SCHMA	ML domain-containing protein	−3.77	0.0001
A0A3Q0KK40_SCHMA	Saposin containing protein	−3.66	0.0034
G4V7C7_SCHMA	Uncharacterized protein	−3.64	0.0002
A0A3Q0KTA0_SCHMA	Uncharacterized protein	−3.60	0.0056
G4LUV4_SCHMA	Subfamily C1A non-peptidase homolog (C01 family)	−3.55	0.0010
A0A3Q0KGW0_SCHMA	Ferritin	−3.40	0.0028
A0A5K4EGY8_SCHMA	Putative annexin	−3.15	0.0014
A0A3Q0KMJ7_SCHMA	Putative programmed cell death protein	−3.15	0.0210

When comparing MF vs. M, 27 and 15 proteins were exclusively identified in MF and M, respectively, and thus could not be quantified. However, the relative abundance of the remaining 700 proteins was not significantly different (Supplementary Figure S3), suggesting that males contribute more to the total MF secretome than females. It is worth noting that for some well-characterized *Schistosoma* spp. vaccine candidates (26) were upregulated in the secretome of females [i.e., A0A5K4F8N6_SCHMA (Sm-TSP-2), Q8MNY2_SCHMA (cathepsin B-1), A0A5K4EE66_SCHMA (thioredoxin glutathione reductase)] or males [i.e., A0A3Q0KIP4_SCHMA, A0A3Q0KD88_SCHMA (paramyosin), FABP_SCHMA (Sm14), G4V6B9_SCHMA (triose phosphate isomerase)]. Furthermore, GST26_SCHMA (glutathione S-transferase 26) was uniquely identified in the secretome from males and two saposins (G4VBU4_SCHMA and A0A3Q0KK40_SCHMA) were also upregulated in the secretome of females. It is also worth highlighting that five different tetraspanins (A0A3Q0KTH5_SCHMA, G4LUN6_SCHMA, A0A5K4FD80_SCHMA, Q86D97_SCHMA and G4LWW2_SCHMA) were uniquely found in the secretome of females, while Sm-TSP-2 was found upregulated in the secretome of females.

### *S. mansoni* Male Adult Worms Secrete Proteins Implicated in Carbohydrate Metabolism, Redox Function, and Cytoskeletal Organization

A protein-protein association (PPA) analysis of proteins uniquely secreted or upregulated in the secretome of *S. mansoni* adult males revealed a strong network of interacting proteins. Approximately 70% of the proteins interacted in one or multiple clusters with other proteins. Whereas, most unique and upregulated proteins interacted together, one cluster of male-unique proteins could be observed (Supplementary Figure S4, circle), which included splicing factors, RNA binding proteins and ribonucleoproteins. Furthermore, a clear cluster of proteins with upregulated abundance in males was associated with glycolysis and gluconeogenesis pathways (Supplementary Figure S4, rectangle).

**Figure 2:**
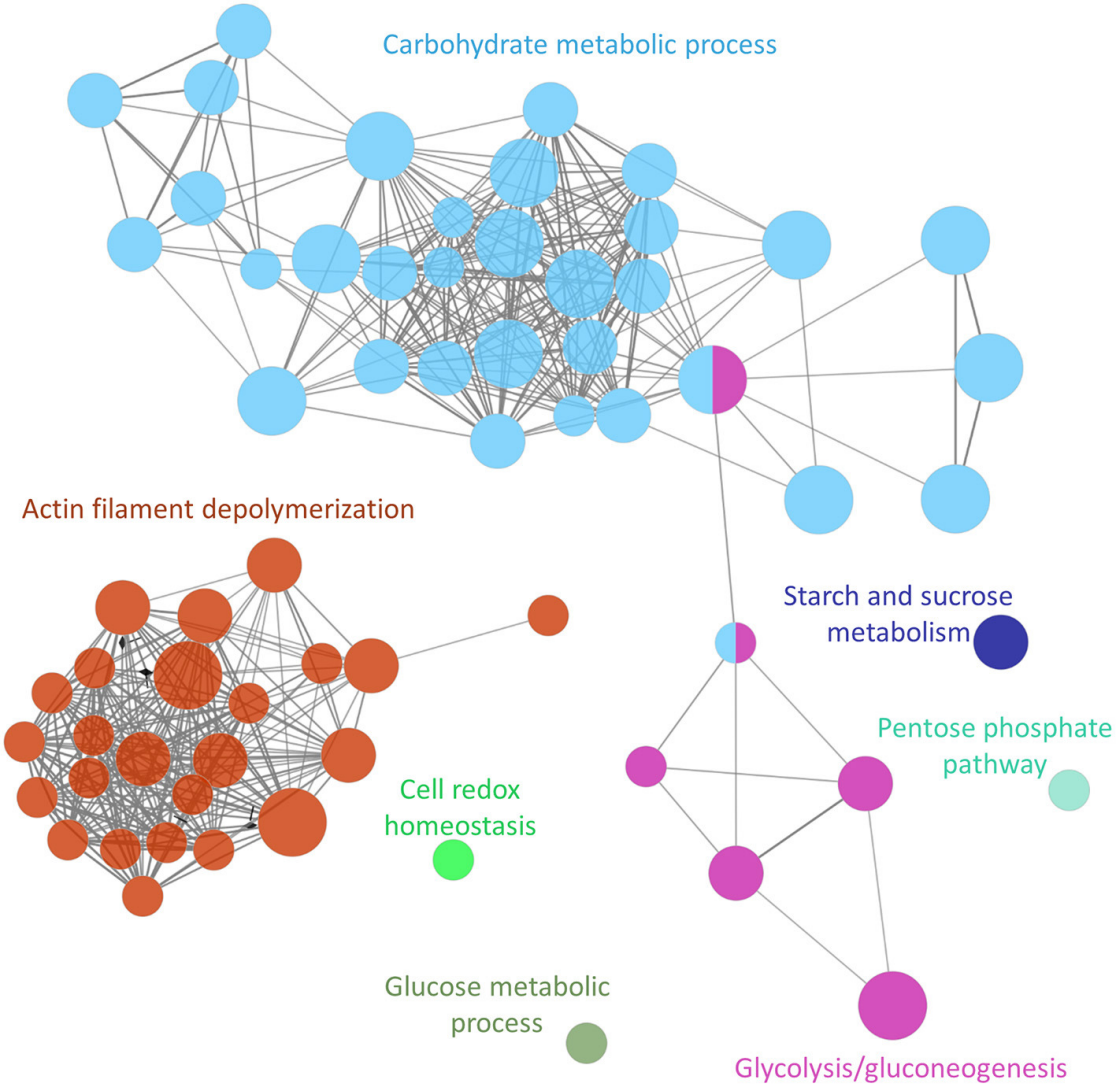
Functional analysis network showing the gene ontology terms and KEGG pathways with the highest significance of proteins uniquely secreted or upregulated in the secretome of the adult *Schistosoma mansoni* male.

The functional analysis showed at least 7 groups (adjusted *P*-value <0.0005) containing 67 non-redundant biological terms (adjusted *P*-value <0.05), including “glucose metabolic process” (GO:0006006), “cell redox homeostasis” (GO:0045454), “pentose phosphate pathway” (KEGG:00030), “starch and sucrose metabolism” (KEGG:00500, “glycolysis/gluconeogenesis” (KEGG:00010), “actin filament depolymerization” (GO:0030042) and “carbohydrate metabolic process” (GO:0005975) (Figure 2; Supplementary Table S3).

The cluster containing the highest number of terms (31 nodes) and proteins (total of 54 proteins) was associated with pathways involved in carbohydrate metabolism (a gene ontology term also associated with the KEGG pathway glycolysis/gluconeogenesis) (Figure 2; Supplementary Table S3). Proteins involved in this pathway included phosphoenolpyruvate carboxykinase, glucose-6-phosphate dehydrogenase and malate dehydrogenase among others. The cluster with the next highest number of terms (24 nodes) and proteins (22 proteins) was associated with a cytoskeleton organization function, and included calponin, paramyosin, filamin, and tubulin.

### *S. mansoni* Female Adult Worms Secrete Proteins With Hydrolase Activity and Cellular Homeostasis

The PPI analysis of proteins uniquely secreted or upregulated in the secretome of the adult female showed that only around 50% of the proteins interacted in one or multiple clusters with other proteins (Supplementary Figure S5). Contrary to what was observed for male schistosomes, uniquely female-secreted proteins or proteins with an upregulated expression in the secretome of females did not cluster together (Supplementary Figure S5).

**Figure 3:**
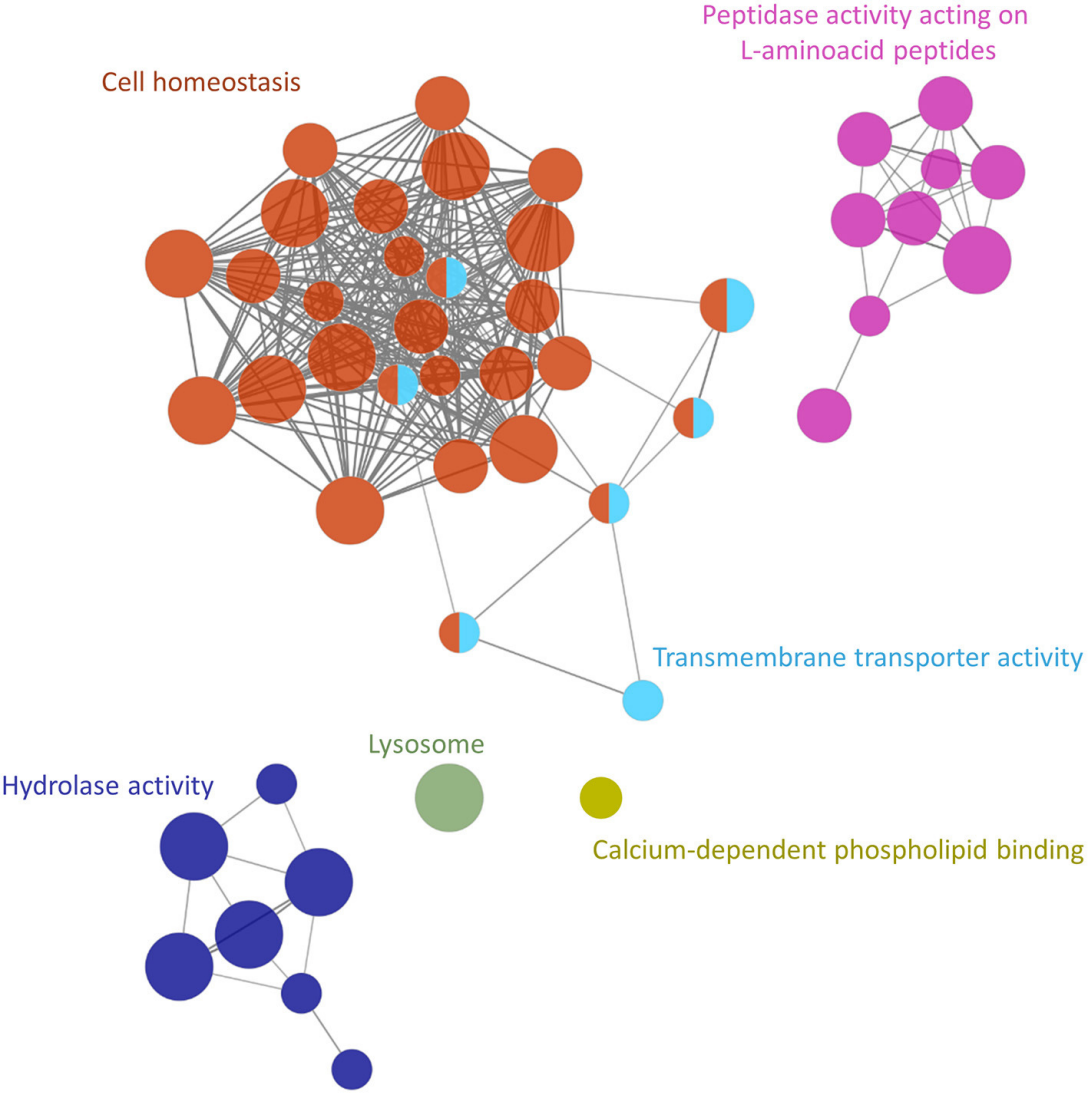
Functional analysis network showing the gene ontology terms and KEGG pathways with the highest significance of proteins uniquely secreted or upregulated in the secretome of the *Schistosoma mansoni* adult female.

The functional analysis showed at least 6 groups (adjusted *P*-value <0.005) containing 54 non-redundant biological terms (adjusted *P*-value <0.05), including “lysosome” (KEGG:04142), “calcium-dependent phospholipid binding” (GO:0005544), “transmembrane transporter activity” (GO:0022857), “hydrolase activity” (GO:0016798), “peptidase activity” (GO:0070011) and “transition metal ion transport” (GO:0000041) (Figure 3; Supplementary Table S4). The cluster with the highest number of terms and proteins was associated with the cellular homeostasis process (28 nodes and 20 proteins) and included ferritin and thioredoxin glutathione reductase (Figure 3; Supplementary Table S4). Additionally, two clusters associated with peptidase activity (9 nodes and 15 proteins) and hydrolase activity (7 nodes and 11 proteins) were also highlighted to be of importance in this analysis (Figure 3; Supplementary Table S4). These two clusters included galactosidase, glucosidase, cathepsin B, cathepsin L, carboxypeptidase, other cysteine- and serine-peptidases, and others (Supplementary Table S4).

### Intersection and Significant Enrichment of Detected ESPs Among Tissue Cluster-Enriched Gene Sets

Wendt et al. recently identified transcriptomic signatures associated with specific tissues in *S. mansoni* using single cell RNA-seq profiling (scRNAseq) (Wendt et al., 2020), with 4,143 genes significantly upregulated in one or more of the 68 molecularly-distinct annotated clusters. Of the 793 ESPs from adult female and male stages of *S. mansoni*, 449 were identified among the cluster-enriched genes from this scRNAseq study. Significant enrichment (after FDR correction) was identified for 24 tissue clusters among all 793 ESPs, nine clusters among the 302 genes either unique or higher in adult female ESPs, and 12 clusters among the 370 genes either unique or higher in adult male ESPs (Table 3; Supplementary Table S7). The 793 total ESPs and the 302 female-specific or overexpressed ESPs were most significantly enriched among the “gut” cluster, with 54.3% and 43.2% of the gut cluster-associated genes being identified in these ESP sets (respectively; *P* <10^−15^ for both comparisons; Supplementary Table S7). This indicated that ESPs from the females are likely secreted from the intestine, whereas the male ESPs contribute to the enrichment of the muscle-associated clusters identified in the combined ESP set (Table 3; Supplementary Table S7).

**Table 3:** Intersection and significant enrichment of ESPs among tissue cluster-enriched gene sets identified from the scRNAseq study of Wendt et al. (2020).

***S. mansoni*** **scRNAseq clusters (Wendt et al.**, **2020****)**		**Number of cluster-enriched genes in ESP protein sets**			**FDR-corrected** ***P***-values for enrichment (binomial dist. test)^*^		
**Cluster identity**	**Number of enriched genes in cluster**	**All 793 proteins detected**	**302 unique or higher in female**	**370 unique or higher in male**	**All 793 proteins detected**	**302 unique or higher in female**	**370 unique or higher in male**
Gut	81	44	35	2	<10^−15^	<10^−15^	–
Muscle 3	239	79	5	57	<10^−15^	–	<10^−15^
Muscle 2	350	95	6	71	<10^−15^	–	<10^−15^
Muscle 7	354	92	5	67	<10^−15^	–	<10^−15^
Muscle 1	317	84	5	58	1.4 ×10^−13^	–	<10^−15^
Muscle 6	387	92	8	62	3.1 ×10^−12^	–	<10^−15^
Muscle 4	365	88	8	62	4.5 ×10^−12^	–	<10^−15^
Muscle 8	374	89	7	62	5.8 ×10^−12^	–	<10^−15^
Muscle 5	331	82	7	54	6.1 ×10^−12^	–	<10^−15^
sm13+	109	33	9	6	4.2 ×10^−7^	0.0026	–
Unknown	227	51	18	13	2.5 ×10^−6^	3.5 ×10^−5^	–
prom2+	104	30	10	8	3.5 ×10^−6^	5.2 ×10^−4^	–
Early tsp-2+	134	35	10	8	4.2 ×10^−6^	0.0028	–
Parenchyma 1	382	73	21	24	4.3 ×10^−6^	7.0 ×10^−4^	–
Tegument 2	235	48	16	9	5.2 ×10^−5^	6.2 ×10^−4^	–
Tegument 1	375	65	16	15	2.8 ×10^−4^	0.037	–
Parenchyma 2	434	73	19	28	2.8 ×10^−4^	0.016	–
Neuron 23	355	61	6	32	5.5 ×10^−4^	–	4.3 ×10^−4^
Early vitellocytes	153	32	6	7	6.4 ×10^−4^	–	–
S1 progeny	126	27	2	7	0.0012	–	–
S1	89	21	3	6	0.0012	–	–
Neuron 17	268	47	6	23	0.0016	–	0.0067
egc+	188	35	7	9	0.0024	–	–
Neuron 20	354	55	7	27	0.0085	–	–
Neuron 18	339	50	5	29	–	–	0.0021
Neuron 21	406	57	6	31	–	–	0.0076

* *“–” Denotes FDR corrected P-values > 0.01*.

## Discussion

Schistosomes are among the most important human helminths in terms of public health, morbidity and mortality. Unlike other trematodes, they are usually found as paired couples, with the female occupying the gynaecophoric canal of the male (Mcmanus et al., 2018). This dioecy distinguishes schistosomes from other human flatworms. Moreover, the sexual dimorphism has biological implications, including the need for pairing to achieve sexual maturity in females (reviewed in Moore et al., 1954), and favoring a division of labor between the muscular male migrating toward oviposition sites and the more delicate, filiform female reaching small vessels to discharge the eggs (Loker and Brant, 2006). Yet despite their importance in male-female communication, only a handful of studies have analyzed in depth the molecules, mediators and receptors involved in these interactions (Armstrong, 1965; Basch and Basch, 1984; Gupta and Basch, 1987; Hall et al., 2011; Chen et al., 2022). In this study, we set out to comprehensively characterize the protein complement of the ESPs from adult stage female and male *S. mansoni* to increase knowledge into the biology of these worms and to augment the repertoire of potential diagnostic and vaccine candidates for schistosomiasis. We cultured adult worms separately for seven days *in vitro* and analyzed the ESPs by mass spectrometry. Although extended incubation times (as well as pairing of adult males and females) have been shown to influence the worms [e.g., loss of mature vitellocytes after 6 days of culture (Wang et al., 2019)], other studies have also cultured worms for 4 days or longer (Hall et al., 2011). A time-series proteomic analysis as well as differences in ESPs in adult worms from single-sex infections would likely be informative with respect to culture conditions and pairing in schistosome development.

Proteins released and secreted by schistosomes, including tegumental proteins and digestive enzymes (in the vomitus Hall et al., 2011) play a key role in host-parasite and male-female interactions, and their expression and secretion is driven by the divergent requirements and functions of both schistosome sexes. For instance, in *S. japonicum* and *S. bovis*, the male schistosome exhibits significantly more proteins in its tegument than the female, both in total number and in unique proteins (Perez-Sanchez et al., 2008; Zhang et al., 2013). Our present findings agree with this assessment and, indeed, were not unexpected given the larger size of the male schistosome. Furthermore, the protein and small RNA content of *S. japonicum*-secreted extracellular vesicles and the expression of phosphoproteins from *S. mekongi* are also sex-dependant (Du et al., 2020). The present results also revealed a gender-specific divergence in the secretome landscape in *S. mansoni*, which, as shown at the transcriptomic level, could be related to the sexual dimorphism of the schistosome (Fitzpatrick et al., 2005; Anderson et al., 2015; Picard et al., 2016). *S. japonicum* female-tegumental proteins are involved in protein glycosylation and lysosome function, while male-tegumental proteins play a role in intracellular signal transduction, regulation of actin filament polymerization, and proteasome core complex (Zhang et al., 2013). Our findings also showed functions related to actin filament depolymerisation and lysosome to be important in male and female-secreted proteins, respectively. While proteins belonging to the lysosome KEGG ontology are a markedly heterogeneous group including cathepsin B peptidases, tetraspanins and glycosidases, proteins related to actin filament depolymerisation play a specific role in cytoskeletal regulation. These results confirm previous transcriptomic studies and support the hypothesis of the role of the male schistosome in physical support of the female to facilitate migration against the flow of the portal circulation toward the mesenteric venules where the schistosome eggs are deposited (Fitzpatrick et al., 2005; Cai et al., 2016; Phuphisut et al., 2018). Indeed, when comparing male-specific and significantly expressed male ESPs with recently published scRNA data, we observed and enrichment of the muscle-associated clusters.

Glucose metabolism is essential in the female schistosome due to the energy requirement to support the production and release of the large number of eggs—around 300 eggs per female per day in *S. mansoni* (Cheever et al., 1994). Glycogen consumption, however, is paramount for other functions such as muscle contraction and tegumental membrane repair, both being significantly enriched among proteins abundant in the adult male vs. the female (Gobert et al., 2003). Earlier transcriptomic investigation revealed that the expression of the glucose transporters *gtp1* and *gtp4* was uninfluenced by schistosome gender (Cai et al., 2016). Here, by contrast, significantly elevated abundance of GTP1 (Smp_012440, Q26579_SCHMA) and other glucose transporters (Smp_105410, G4VC44_SCHMA) was evident in the female *S. mansoni*, likely the result of an increased expression in the tegument. These discrepancies may in part be explained since transcriptomic analysis was performed on whole worms whereas the present study focused on the ESPs. Other glycolytic enzymes such as aldolase (Smp_042160.1, ALF_SCHMA) and glycerol-3-phosphate dehydrogenase (G3PDH, Smp_030500.1, C4Q5J8_SCHMA) were more abundantly secreted by the male. Our findings conformed with previous reports of higher consumption of glucose and importance of glycogen storage in the male worm, which could reflect the muscular effort involved in transporting the female through the portal vasculature, and the need by the female to transport these metabolites from the male (Skelly et al., 2014). Notably from the viewpoint of infection control, aldolase and G3PDH have been a focus of vaccines and other intervention targets (Dessein et al., 1988; Goudot-Crozel et al., 1989; Tallima et al., 2017).

In addition, the findings indicate that hydrolases and peptidases play an important role in the biology of female schistosomes. The hydrolase grouping includes lipases, phosphatases, and glycosidases among others. In the case of the female schistosome, several glycosidases were found more abundantly secreted, including beta-glucosidase (Smp_043390, A0A3Q0KF32_SCHMA), alpha-galactosidase (Smp_089290, G4VLE3_SCHMA), and several alpha-amylases. Furthermore, it has been suggested that hydrolases released by the schistosome egg contribute to the transit of the egg and the circumoval granuloma across the intestinal wall and also with the nutritional requirements of the embryo (Cesari et al., 2000). Despite our findings, we cannot rule out that hydrolases detected here could have been secreted by eggs when released by females *in vitro* and may not originate from the adult female. Other peptidases including cathepsins participate in invasion of the skin by the cercaria (Dvorak et al., 2008), hemoglobin degradation by the adult stage (Gotz and Klinkert, 1993; Dalton et al., 1995), and egg hatching (Rinaldi et al., 2009), and have profound immunogenic properties (Soloviova et al., 2019). Females secreted several cathepsins more abundantly than males, including SmCB1 (Q8MNY2_SCHMA), which is known to be secreted from the gut of schistosomes and to contribute to Th2 polarization responses (De Oliveira Fraga et al., 2010). This enzyme has been validated as a potent anti-schistosome chemotherapeutic target (Abdulla et al., 2007; Jilkova et al., 2021).

A number of other well-characterized vaccine candidates were upregulated in the secretome of the male *S. mansoni* adult, i.e., Sm14 (Smp_095360.1, FABP_SCHMA), GST26 (Smp_163610.1, GST26_SCHMA), GST28 (Smp_054160.1, GST28_SCHMA), three paramyosin isoforms (Smp_046060.1, A0A3Q0KFC2_SCHMA; Smp_085540.6, A0A3Q0KIP4_SCHMA; Smp_021920.3, A0A3Q0KD88_SCHMA), and in the secretome of the female, i.e., Sm-TSP-2 (Smp_335630.1, A0A5K4F8N6_SCHMA), thioredoxin glutathione reductase (A0A5K4EE66_SCHMA), cathepsin B1 (Smp_103610.1, Q8MNY2_SCHMA) and several saposins (Smp_194910.1, G4VHH1_SCHMA; Smp_130100.1, G4VBU4_SCHMA, Smp_105450.1, A0A3Q0KK40_SCHMA). Most tetraspanins identified were significantly upregulated in the secretome of female worms. Sm-TSP-2 formulated with glucopyranosyl lipid adjuvant has proven safe in a phase I trial (Keitel et al., 2019), and a homolog in *S. haematobium* (Sh-TSP-2) shows efficacy in a heterologous mouse model of schistosomiasis (Mekonnen et al., 2020). Furthermore, tetraspanins represent confirmed diagnostic markers of urogenital schistosomiasis (Pearson et al., 2021; Mekonnen et al., 2022). Notably, we observed five additional tetraspanins uniquely present in the female ESP. Given the expanding reputation of tetraspanins as diagnostic antigens for detection of infection with *S. haematobium* (Pearson et al., 2021; Mekonnen et al., 2022), these homologs from *S. mansoni* might be worthy of consideration for serodiagnosis. It is also noteworthy that both GST26 and GST28 were upregulated in the secretome of the male. Although GSTs have been extensively investigated as vaccine candidates (Riveau et al., 1998), recent reports reveal a lack of efficacy against urogenital schistosomiasis (Riveau et al., 2018). Intriguingly, tetraspanins (including Sm-TSP-2) and GSTs have been identified in the extracellular vesicles secreted by *S. mansoni* adult worms (Sotillo et al., 2016). Although beyond the scope of this report, it would be valuable to analyse the contribution of each sex to the secretion of extracellular vesicles and to determine their composition and biochemical profiles.

We have additionally identified 75 and 237 proteins uniquely secreted by female and male worms, respectively. Whereas, these proteins might reflect the gender-specific biology, they also likely contribute to male-female communication. Recent reports have highlighted the secretion by male schistosomes of a specific small molecule (ß-alanyl-tryptamine) that is key for the development and laying of eggs by females (Chen et al., 2022) (ß -alanyl-tryptamine is a diminutive peptide of ~300 Daltons in mass which would not have been retained in our samples during centrifugation which employed a 3 kDa cutoff membrane). Furthermore, in *S. japonicum*, biogenic amine neurotransmitters are implicated in male-female sexual communication (Wang et al., 2017). Based on the present findings, we posit that the development of drugs interrupting male-female communication could lead to novel and effective control measures.

To conclude, a broader coverage of the *S. mansoni* ESP profile, and deeper knowledge of the secretome contributes to our understanding of schistosome biology, and thus these new findings provide new information including leads for the development of novel vaccine strategies. Moreover, identification of the most abundantly secreted proteins of the schistosome sexes should enhance analysis of the regulatory elements and motifs that control the expression of the corresponding genes. This will be of assistance in the development of transgenic schistosomes that over-express endogenous proteins, or even secrete foreign proteins. Access by the field to transgenic schistosomes that (conditionally) secrete reporters, model antigens, and other informative gene products, along with advances in human challenge models (Langenberg et al., 2020) can be expected to hasten progress in the immunobiology and pharmacology of these flukes (Hoffmann et al., 2014; Zamanian and Andersen, 2016; Mcveigh and Maule, 2019; Douglas et al., 2021; Quinzo et al., 2022).

## Data Availability Statement

Mass spectrometry data along with the identification results have been deposited in the ProteomeXchange Consortium via the PRIDE partner repository with the dataset identifier PXD030699.

## Ethics Statement

The animal study was reviewed and approved by Institutional Animal Care and Use Committee (IACUC) at GWU.

## Author Contributions

VM, EK, WI, PB, and JS designed the experiments. EK, MM, BR, BB, AL, PB, and JS analyzed the data. JS, EK, VM, and PB drafted the manuscript with input from all the co-authors. WI, EK, and VM contributed the helminth materials. JS performed mass spectrometry focused analysis. JS, AL, and PB supervised the project. JS, AL, MM, PB, and BB arranged the funding. All authors read and approved the final draft.

## Funding

The findings were obtained from work partially supported by the Defense Advanced Research Projects Agency (DARPA) and Naval Information Warfare Center Pacific (NIWC Pacific), under Contract No. N66001-21-C-4013 (Approved for Public Release, Distribution Unlimited).

## Author Disclaimer

The views, opinions, and/or findings expressed are those of the authors and should not be interpreted as representing the official views or policies of the Department of Defense or the U.S. Government.

## Conflict of Interest

BB was employed by Charles River Analytics, Inc. The remaining authors declare that the research was conducted in the absence of any commercial or financial relationships that could be construed as a potential conflict of interest.

## Publisher's Note

All claims expressed in this article are solely those of the authors and do not necessarily represent those of their affiliated organizations, or those of the publisher, the editors and the reviewers. Any product that may be evaluated in this article, or claim that may be made by its manufacturer, is not guaranteed or endorsed by the publisher.

## Supplementary Material

Supplementary Figure S1 Data normalization using medians of summed intensities after label-fere quantitative analysis.

Supplementary Figure S2 Power calculation and false discovery rate of the label-free quantitative analysis performed using MSstats.

Supplementary Figure S3 Volcano plot of *Schistosoma mansoni* secreted proteins from male vs male-female (mix). Statistically significant differences were not apparent.

Supplementary Figure S4 Protein-protein association network of all proteins uniquely present or with significantly higher abundance in the secretome of the adult *S. mansoni* male.

Supplementary Figure S5 Protein-protein association network of all proteins uniquely present or with a significantly upregulated expression in the secretome of the adult *S. mansoni* female.

Supplementary Table S1 MSstats input file containing unique and razor peptides generated by MSFragger.

Supplementary Table S2 Label-free quantitative analysis of male vs. Female secreted proteins.

Supplementary Table S3 Functional analysis of differentially abundant male secreted proteins using ClueGO.

Supplementary Table S4 Functional analysis of differentially abundant female secreted proteins using ClueGO.

Supplementary Table S5 Corresponding Uniprot and *Schistosoma mansoni* genome codes and annotations for the GO/KEGG terms of proteins uniquely secreted or upregulated in the secretome of *S. mansoni* adult males.

Supplementary Table S6 Corresponding Uniprot and *Schistosoma mansoni* genome codes and annotations for the GO/KEGG terms of proteins uniquely secreted or upregulated in the secretome of *S. mansoni* adult females.

Supplementary Table S7 Proteins secreted by male or female *Schistosoma mansoni* adult worms intersecting and significantly enriched among gut-enriched gene sets identified from a previous scRNAseq study [1].
