# Supplementary figures and images for: Differential Excretory/Secretory Proteome of the Adult Female and Male Stages of the Human Blood Fluke, Schistosoma mansoni
Source: Front Parasitol. 2022 Jul 18;1:950744. doi: 10.3389/fpara.2022.950744 (PMC11732030; doi:10.3389/fpara.2022.950744)

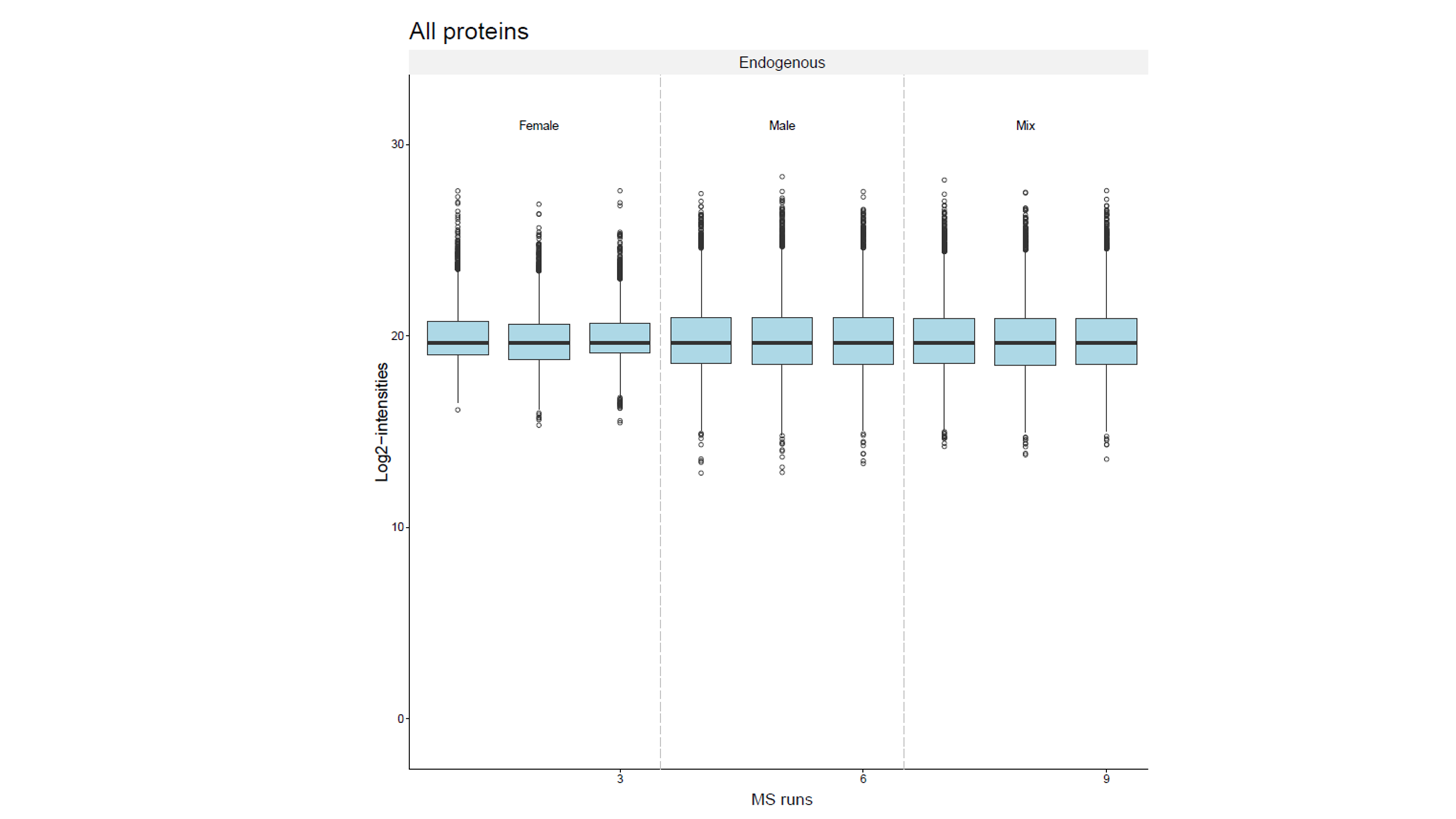

Supplement: Supplementary Figure S1 — Data normalization using medians of summed intensities after label-fere quantitative analysis. [file Image_1.TIF]

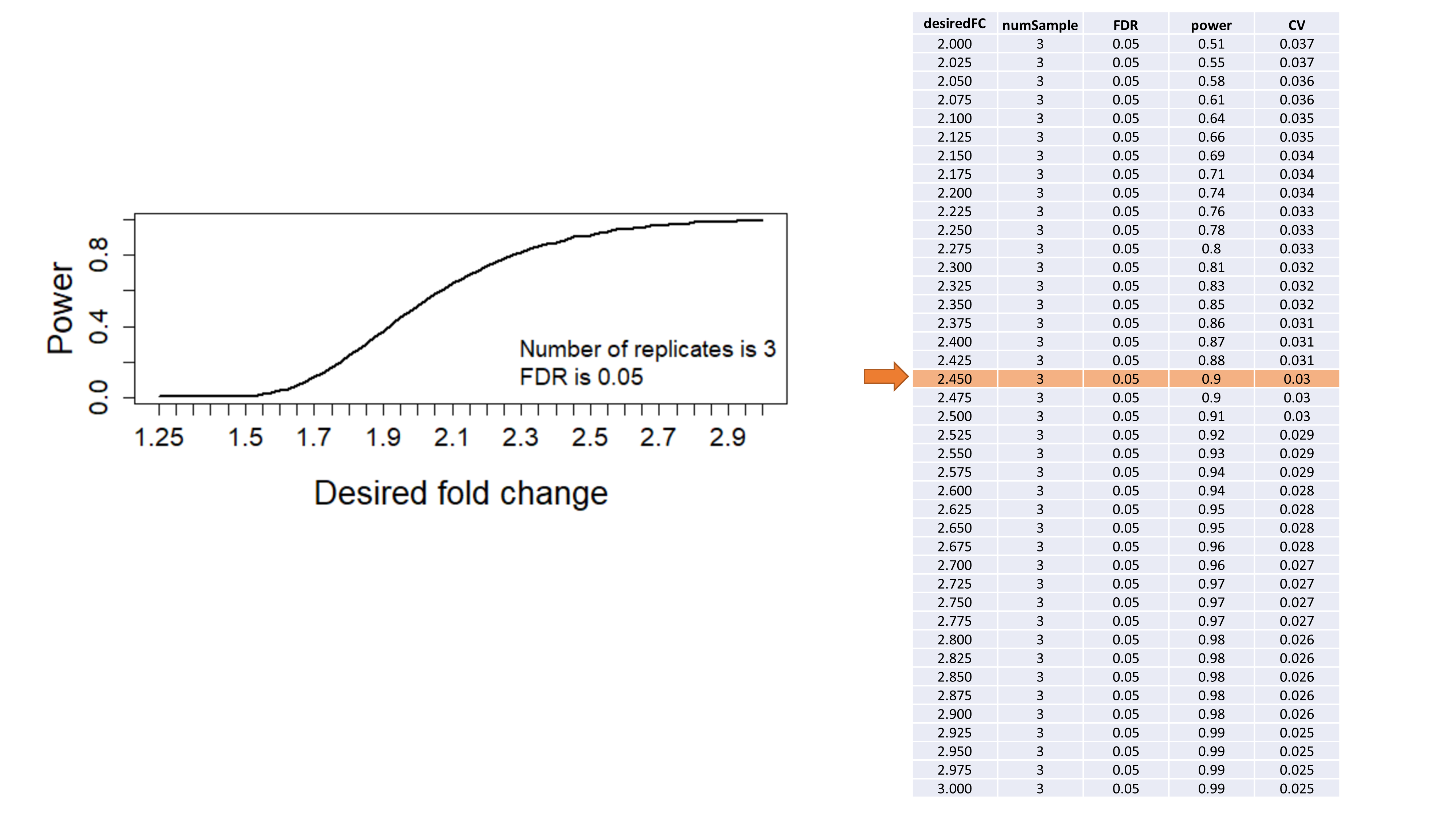

Supplement: Supplementary Figure S2 — Power calculation and false discovery rate of the label-free quantitative analysis performed using MSstats. [file Image_2.TIF]

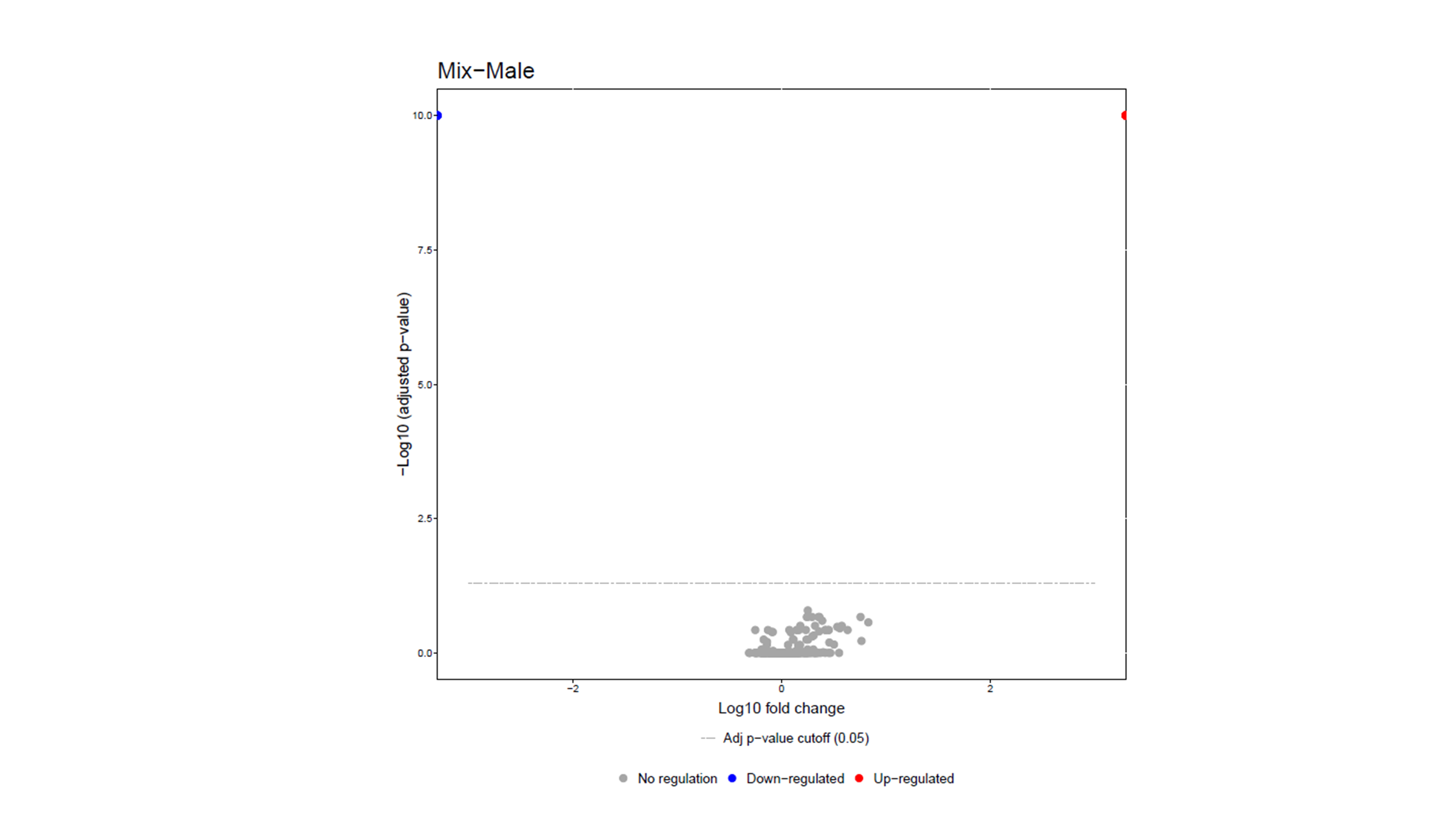

Supplement: Supplementary Figure S3 — Volcano plot of Schistosoma mansoni secreted proteins from male vs male-female (mix). Statistically significant differences were not apparent. [file Image_3.TIF]

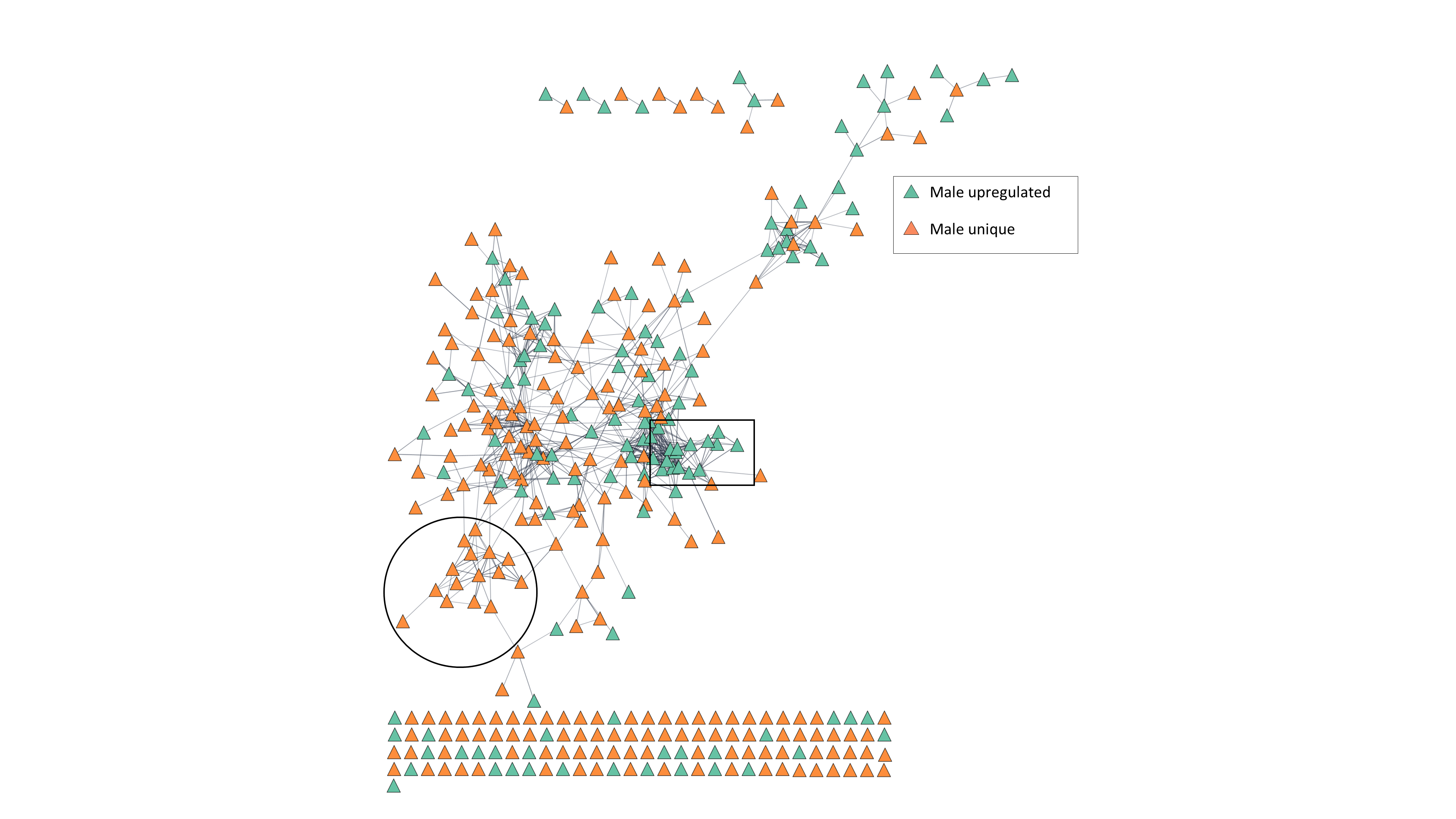

Supplement: Supplementary Figure S4 — Protein-protein association network of all proteins uniquely present or with significantly higher abundance in the secretome of the adult S. mansoni male. [file Image_4.TIF]

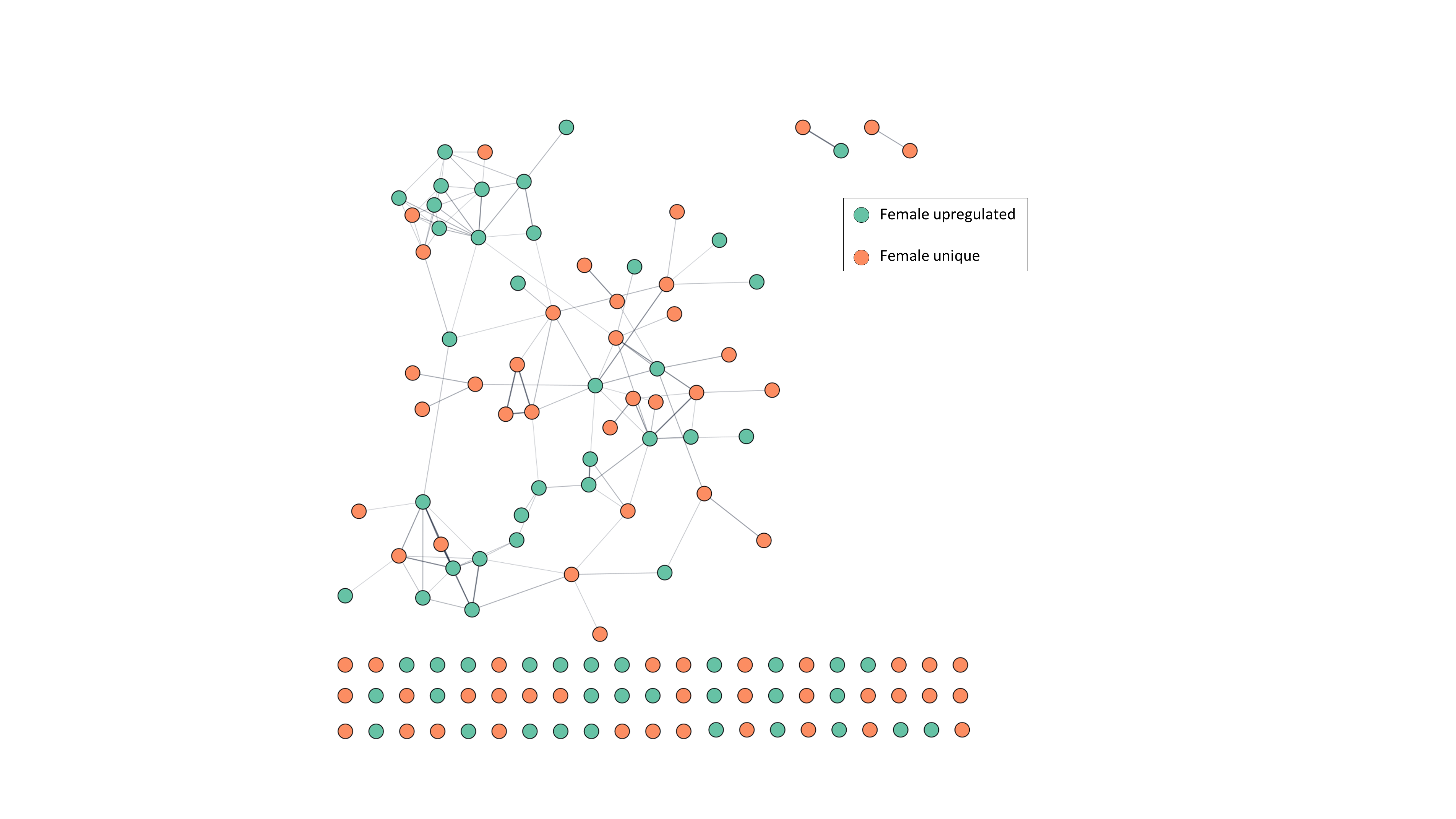

Supplement: Supplementary Figure S5 — Protein-protein association network of all proteins uniquely present or with a significantly upregulated expression in the secretome of the adult S. mansoni female. [file Image_5.TIF]
